# Relationship between PD-L1 expression, CD8+ T-cell infiltration and prognosis in intrahepatic cholangiocarcinoma patients

**DOI:** 10.1186/s12935-021-02081-w

**Published:** 2021-07-12

**Authors:** Min Deng, Shao-Hua Li, Xu Fu, Xiao-Peng Yan, Jun Chen, Yu-Dong Qiu, Rong-Ping Guo

**Affiliations:** 1 Department of Liver Surgery, Sun Yat-Sen University Cancer Center, 651 Dongfeng East Road, Guangzhou, China; 2 State Key Laboratory of Oncology in South China, Guangzhou, China; 3 Collaborative Innovation Center for Cancer Medicine, Guangzhou, China; 4 Department of Hepatopancreatobiliary Surgery, Nanjing Drum Tower Hospital, The Affiliated Hospital of Nanjing University Medical School, 321 Zhong-Shan Road, Nanjing, Jiangsu China; 5 Department of Pathology, Nanjing Drum Tower Hospital, The Affiliated Hospital of Nanjing University Medical School, Nanjing, China

**Keywords:** Intrahepatic, Cholangiocarcinoma, Programmed death-ligand 1, CD8+ T cell, Prognosis

## Abstract

**Background:**

Programmed death- ligand 1 (PD-L1) seems to be associated with the immune escape of tumors, and immunotherapy may be a favorable treatment for PD-L1-positive patients. We evaluated intrahepatic cholangiocarcinoma (ICC) specimens for their expression of PD-L1, infiltration of CD8+ T cells, and the relationship between these factors and patient survival.

**Methods:**

In total, 69 resections of ICC were stained by immunohistochemistry for PD-L1, programmed death factor-1 (PD-1), and CD8+ T cells. CD8+ T-cell densities were analyzed both within tumors and at the tumor-stromal interface. Patient survival was predicted based on the PD-L1 status and CD8+ T-cell density.

**Results:**

The expression rate of PD-L1 was 12% in cancer cells and 51% in interstitial cells. The expression rate of PD-1 was 30%, and the number of CD8+ T-cells increased with the increase of PD-L1 expression (p < 0.05). The expression of PD-L1 in the tumor was correlated with poor overall survival(OS) (p = 0.004), and the number of tumor and interstitial CD8+ T-cells was correlated with poor OS and disease-free survival (DFS) (All p < 0.001).

**Conclusions:**

The expression of PD-L1 in the tumor is related to poor OS, and the number of tumor or interstitial CD8+ T-cells is related to poor OS and DFS. For patients who lose their chance of surgery, PD-L1 immunosuppressive therapy may be the focus of future research as a potential treatment.

## Background

Intrahepatic cholangiocarcinoma (ICC) is the second primary malignant tumor of the liver, originating from the intrahepatic bile duct [1–3]. ICC is characterized by a poor prognosis and various etiological factors [4]. In recent years, epidemiological studies [5, 6] have shown ethnic and regional differences and an increasing trend in the incidence of ICC. The etiology of ICC is mainly associated with basic biliary diseases, such as bile duct stones and primary sclerosing cholangitis [5, 7, 8]. Recent studies have reported an association between ICC and chronic viral hepatitis, including hepatitis B virus (HBV) and hepatitis C virus (HCV) infections [8, 9]. At present, radical surgery is still the only curative therapy for ICC [10–12]. However, most patients have few opportunities to receive radical resection because of the lack of early symptoms. Other remedies, such as radiotherapy, chemotherapy, and targeted therapy, are lack of efficacy [13–16]. Thus, finding an effective treatment for ICC patients, especially for inoperable ICC patients, is significant.

Among the most promising approaches to activating therapeutic anti-tumor immunity is the blockade of immune checkpoints. Evidence [17–20] suggests that programmed death-ligand-1 (PD-L1), an immune checkpoint ligand, represses anti-tumor immunity through its interaction with the programmed death factor 1 (PD-1) receptor of T lymphocytes in various tumors. PD-1 is a transmembrane glycoprotein of the immunoglobulin B7-CD28 family, expressed by activated T cells, natural killer cells, etc. PD-L1, one of the essential PD-1 ligands, can be expressed on the surface of stromal and cancer cells. In chronic inflammation, PD-L1 is upregulated, and its binding to PD-1 induces T-cell exhaustion, thus preventing autoimmunity development. If abundant PD-L1, secreted by cancer cells, binds to PD-1 in the tumor, T cells are directly inactivated. Therefore, the immune mechanisms associated with PD-1/PD-L1 are considered critical points for tumor immune escape [17, 18].

Some inhibitors targeting PD-1/PD-L1 have also been shown to improve the prognosis of several malignancies such as melanoma [21], gastric carcinoma [22], and non-small cell lung cancer [23]. Attention has currently been focused on the identification of predictive biomarkers for the selection of patients for treatment. The question of whether the expression of PD-1/PD-L1 correlates with treatment outcomes has been addressed in most of pivotal trials, but the answer is still unclear. Some of these inconsistencies are assay-related, with no general consensus regarding which antibody to use, which cells to stain, and what cut-off value to choose. In addition, there is a close relationship between the density of CD8+ T-cells and the tumor immune environment. A decreased density of CD8+ T-cells always indicates tumor immune resistance[24]. The density of CD8+ T-cells, the expression of PD-L1, and the tumor progression and prognosis are closely related in colorectal cancer [25], gastric adenocarcinoma [26], and hepatocellular carcinoma (HCC) [27]. Studies have demonstrated that the expression of PD-L1 in HCC is significantly higher than that in liver cirrhosis or chronic hepatitis [28, 29]. Moreover, HCC patients with high PD-L1 expression have a significantly worse prognosis. For advanced HCC patients, combination immunotherapy with sorafenib and nivolumab induces effective natural killer cell responses, resulting in a better prognosis than that for a single-drug treatment [30]. However, information on the expression of PD-1/PD-L1 in ICC is very limited, and its relationship with clinical and histopathological features of this cancer remains unknown.

Unlike those in HCC, cancer cells in ICC are surrounded by an abundant fibrous stroma. It is important to evaluate the expression of PD-1 and PD-L1 in various cells in ICC. In this research, we aimed to examine the PD-L1 and PD-1 expression in 69 surgically resected ICC specimens from patients with various underlying risk factors and to determine its relationship with clinical parameters and pathological features, as well as with the density of CD8+ T-cells and patient survival.

## Materials and methods

### Patients and specimens

A total of 121 ICC patients underwent surgical treatment at our hospital. Patients who met the inclusion criteria were enrolled in this study. Finally, 69 patients with resected ICCs were selected for this retrospective analysis.

The inclusion criteria including: (1) Radical resection was performed at the first treatment; (2) Confirmed by pathology; (3) Complete clinicopathological data.

Consents for operation and research were obtained in all individuals before the surgical resection. This study was approved by the Medical Ethics Committee of the Nanjing Drum Tower Hospital.

### Collection of clinical and pathological data

Medical records of all patients were reviewed. Clinical and pathological information was tabulated, including the sex, age, virological markers, tumor markers, and hepatic function indicators.

Tumor TNM staging was performed using the 8th edition of the American Joint Committee on Cancer (AJCC) Staging Manual.

Pathological changes such as inflammation and cirrhosis in the uninvolved adjacent hepatic or bile duct tissue were also reviewed microscopically and analyzed according to the Batts–Ludwig stages of inflammation and fibrosis.

### Immunohistochemical staining

Representative 4-μm serial sections of a tumor were prepared for immunohistochemistry from 10% formalin-fixed, paraffin-embedded tissue blocks. ICC tissues and non-tumor tissues for immunostaining were following deparaffinized, rehydrated, and antigen retrieval. All slides were baked at 60 °C overnight, subjected to microwave retrieval in citrate buffer, and exposed to 3% hydrogen peroxide for 10 min to block the endogenous peroxidase activity. Immunohistochemistry was performed using anti-PD-1 (1:300, Proteintech), PD-L1 (1:500, Proteintech), and CD8 (1:500, Abcam) as the primary antibodies in a humidified chamber at 4 °C overnight, followed by incubation with an anti-mouse peroxidase-conjugated secondary antibody (1:100, Dako) at 37 °C for 30 min. Both negative (without the primary antibody) and positive controls were included in each run. DAB detection kit (50:1, Dako) was used to visualize slides, and expression status was assessed by light microscopy. All the sections were analyzed under a Leica DM 2000 optical microscope (Leica Microsystems, Wetzlar, Germany), and microphotographs were collected using a Leica DFC320 digital camera (Leica).

Specimens were defined as positive if PD-L1 was expressed in ≥ 2% of cancer cells, as well as if PD-L1 and PD-1 were expressed in ≥ 2% of stromal cells.

In this study, CD8+ T cells were counted in the tumor and tumor stroma under a 40× magnification; the number of CD8+  T cells was counted in 3 specific regions, and the average value was calculated. To count CD8+ lymphocytes, three positive regions were selected in each slice, and the numbers of CD8+ tumor-infiltrating lymphocytes (TILs) and CD8+ non-tumor-infiltrating lymphocytes (NILs) were counted each time (400 times). The mean number was used as the lymphocyte count. Based on the numbers of CD8+ T-cells in the tumor and stroma, specimens were categorized into high-density (≥ 40/Hp) and low-density (< 40/Hp) subgroups.

### Statistical analysis

All statistical analysis in this study was analyzed with SPSS 17.0 software (SPSS Inc, Chicago, Illinois, US). Data are shown as “mean ± standard deviation” and “median (range)”, as appropriate. The Student t-test and one-way analysis of variance (ANOVA) were performed to analyze differences between two groups. The Chi-square or Fisher's exact test was utilized for comparison of ratios. Patient’s survival was estimated by the Kaplan–Meier method with a log-rank test. A Cox proportional hazard model was to evaluate risk factors of survival. The hazard ratio (HR) and its 95% confidence interval (CI) were calculated. Differences were considered to be statistically significant when *p* < 0.05.

## Results

### Clinicopathological features, expression of PD-L1 and PD-1 in ICC tissues

**Fig. 1:**
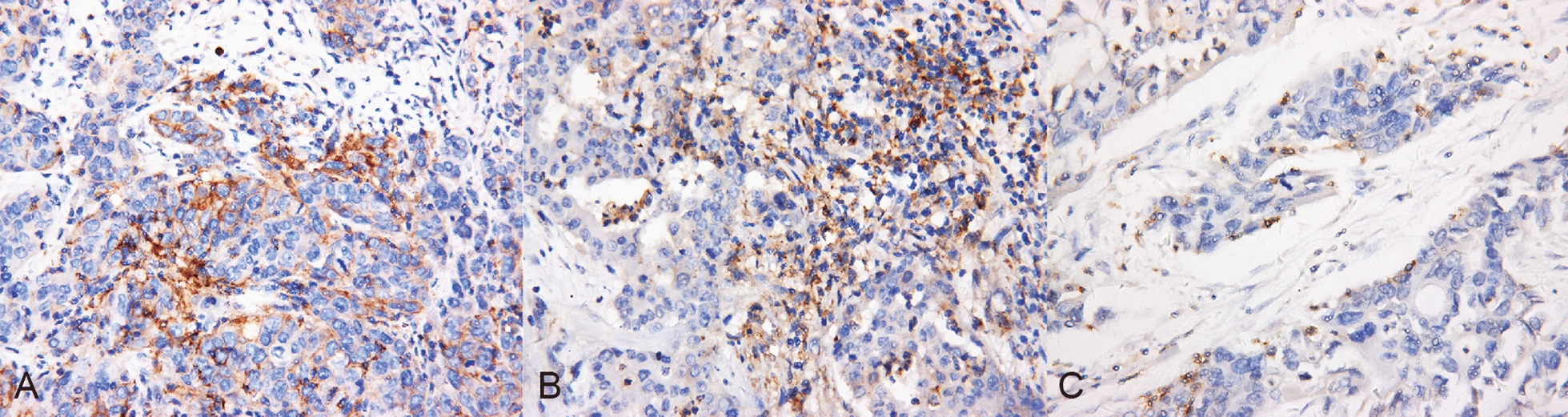
Representative immunohistochemical staining of PD-L1 and PD-1 in Intrahepatic Cholangiocarcinoma tissues. **A** Immunostaining for PD-L1 expression in cancer cells; **B** Immunostaining for PD-L1 expression in interstitial cells; **C** Immunostaining for PD-1 expression on TILs. Shown at ×400 original magnification

Among the 69 ICC patients, 40 were males, and 29 were females. The age ranged from 34 to 81 years old, with an average age of 60 years. The diameter of the tumor ranged from 0.5 to 12 cm. A single tumor was found in 78.3% (54/69) of the patients, and multiple tumors were found in 21.7% (15/69) of the patients. Positive results of PD-L1 expression are shown in Fig. 1. Among the patients, 11.6% (8/69) were positive for PD-L1 expression in cancer cells, while in 50.1% (35/69) of the patients, PD-L1 was expressed in interstitial cells. The rate of PD-1 expression in tumor lymphocytes was 30.4% (21/69). The relationship between clinicopathological features and PD-L1 expression, PD-1 expression are shown in Tables 1 and 2, respectively.

**Table 1:** Relationship between clinical characteristics and PD-L1 and PD-1 expression in intrahepatic cholangiocarcinoma

	n	PD-L1 expression				PD-1 expression	
		Cancerous cell (+)	*p* value	Stromal cells (+)	*p* value	Lymphocyte (+)	*p* value
Total number	69	8 (11.6%)		35 (50.7%)		21 (30.4%)	
Age, years; median (range)	60 (34–81)	63 (46–79)	0.499	63 (43–81)	0.025	60 (50–76)	0.984
Gender			0.266		0.264		0.006
Male	40	3 (7.5%)		18 (45%)		7 (17.5%)	
Female	29	5 (17.2%)		17 (58.6%)		14 (48.3%)	
HBV infection	28		0.337		0.555		0.781
HBsAg, positive		5 (17.9%)		13 (46.4%)		8 (28.6)	
HBsAg, negative		3 (7.3%)		22 (53.7%)		13 (31.7%)	
CA19-9 > 39.9U/ml	38		0.494		0.726		0.177
CA19-9, positive		3 (7.9%)		20 (52.6%)		9 (23.7%)	
CA19-9, negative		5 (16.1%)		15 (48.4%)		13 (41.9%)	
TB > 20.5umol/L	13		1.0		0.803		0.33
TB, positive		2 (15.4%)		7 (53.8%)		2 (15.4%)	
TB, negative		6 (10.7%)		28 (50%)		19 (33.9%)	
DB > 6.8umol/L	10		0.865		0.187		0.974
DB, positive		1 (10%)		7 (70%)		3 (30%)	
DB, negative		7 (11.9%)		28 (47.5%)		18 (30.5%)	
ALT > 40U/L	16		0.644		0.184		0.916
ALT, positive		3 (18.8%)		11 (68.8%)		5 (31.3%)	
ALT, negative		5 (9.4%)		24 (45.3%)		16 (30.2%)	
AST > 40U/L	18		0.724		0.305		0.776
AST, positive		3 (16.7%)		11 (61.1%)		5 (27.8%)	
AST, negative		5 (9.8%)		24 (47.1%)		16 (31.4%)	
γ-GT > 35U/L	48		0.957		0.056		0.824
γ-GT, positive		5 (10.4%)		28 (58.3%)		15 (31.3%)	
γ-GT, negative		3 (14.3%)		7 (33.3%)		6 (28.6%)	

*HBV* hepatitis B virus, *HBsAg* hepatitis B surface antigen, *CA19-9* Carbohydrate antigen 19–9, *TB* total bilirubin, *DB* direct bilirubin, *ALT* alanine transaminase, *AST* aspartate transaminase, *γ-GT* gamma-glutamyl transpeptidase

**Table 2:** Relationship between pathological characteristics and PD-L1 and PD-1 expression in intrahepatic cholangiocarcinoma

	n	PD-L1 expression				PD-1 expression	
		Cancerous cell (+)	*p* value	Stromal cells (+)	*p* value	Lymphocyte (+)	*p* value
Tumor size, cm			0.806		0.9		0.034
> 5 cm	33	3 (9.1%)		17 (51.5%)		6 (18.2%)	
≤ 5 cm	36	5 (13.9%)		18 (50%)		15 (41.7%)	
Tumor number			0.812		0.348		0.499
Single	54	6 (11.1%)		29 (53.7%)		18 (33.3%)	
Multiple	15	2 (13.3%)		6 (40%)		3 (20%)	
Gross types			0.186		0.348		0.22
MF	54	8 (14.8%)		29 (53.7%)		14 (25.9%)	
Non-MF	15	0 (0)		6 (40%)		7 (46.7%)	
Differentiation			0.043		0.308		0.408
Well		0 (0)		1 (2.8%)		1 (4.8%)	
Moderately		5 (63%)		29 (82.9%)		19 (90.4%)	
Poorly or undifferentiated		3 (37%)		5 (14.3%)		1 (4.8%)	
CLC or cICC	14	3 (21.4%)	0.412	9 (64.3%)	0.256	3 (21.4%)	0.621
MVI	25	4 (16%)	0.638	0 (0)	0.199	4 (16%)	0.005
Neural invasion	69	8 (11.6%)	0.227	35 (50.7%)	0.988	21 (30.4%)	1.0
TNM stage			0.245		0.429		0.282
I	28	1 (3.6%)		6 (21.4%)		8 (28.6%)	
II	25	5 (20%)		13 (52%)		6 (24%)	
III	5	1 (20%)		2 (40%)		1 (20%)	
IV	11	1 (9.1%)		6 (54.5%)		6 (54.5%)	
Breakthrough the liver	12	1 (8.3%)	0.698	6 (50%)	0.956	3 (25%)	0.916
Grade of inflammation			0.961		0.504		0.853
G0,G1or G2	60	7 (11.7%)		29 (48.3%)		19 (31.7%)	
G3 or G4	9	1 (11.1%)		6 (66.7%)		2 (22.2%)	
Fibrosis/cirrhosis			0.012		0.77		0.686
S0,S1 or S2	59	4 (6.78%)		29 (49.2%)		19 (32.2%)	
(S3 or S4)	10	4 (40%)		6 (60%)		2 (20%)	
CD8 density/Hp							
CD8+ TIL ≥ 40	4	4 (100%)	< 0.0001	4 (100%)		1 (25%)	
CD8+ NIL ≥ 40	19	6 (31.6%)	< 0.0001	13 (68.4%)		8 (42.1%)	

*MF* mass-forming, *CLC* Cholangiolocellular Carcinoma, *cICC*conventional Intrahepatic Cholangiocarcinoma, *MVI* microvascular invasion

### Relationship between clinicopathological characteristics and PD-L1/PD-1 expression in intrahepatic cholangiocarcinoma

**Fig. 2:**
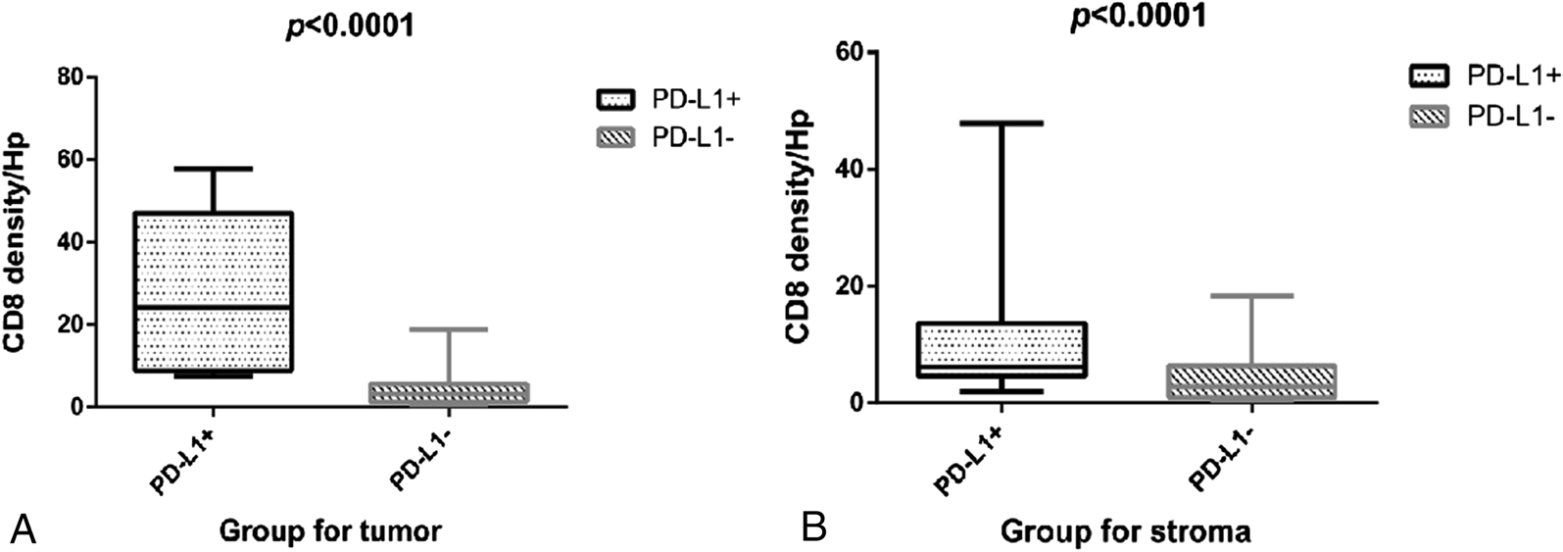
Programmed death-ligand 1 (PD-L1) expression in tumor cells and the stroma increases with the increase of CD8+ TIL densities at each site. The CD8+ TIL densities in ICC were divided by quartiles into low and high. **A** Relation between PD-L1 expression and the number of CD8+ TILs in the tumor. **B** Relation between PD-L1 expression and the number of CD8+ TILs in the stroma

As shown in Table 2, lymphocytes exhibited significantly higher expression of PD-1 in smaller-size tumors (tumor size ≤ 5 cm) than in larger-size ones (tumor size > 5 cm) (*p* = 0.034). Cancerous cells exhibited significantly higher expression of PD-L1 in poorly and moderately differentiated tumors (8/8) than in well-differentiated ones (23/61) (*p* = 0.043). PD-1-positive tumors (4/25) were more frequent than PD-1-negative tumors in patients with liver microvascular tumor thrombosis (*p* = 0.05). Cancer cells exhibited significantly higher expression of PD-L1 (*p* = 0.012) in patients with severe fibrosis (S3 or S4) than in those with weak fibrosis (S0, S1, or S2). The average numbers of CD8+ T-cells in PD-L1-positive tumors (36.3) were significantly higher than those in PD-L1-negative tumors (9.0) (*p* = 0.0001). In addition, the average number of CD8+ T cells in the PD-L1-positive stroma (15.8) was significantly higher than that in the PD-L1-negative stroma (8.4) (*p* = 0.0298). Moreover, the relationship between CD8+ T cell counts and PD-L1 expression in tumor and stroma are shown in Fig. 2. CD8+ T cell counts increased with the increase of PD-L1 expression both in the tumor (*p* < 0.0001) and stroma (*p* < 0.0001). The other results were not statistically significant.

### Prognosis

**Fig. 3:**
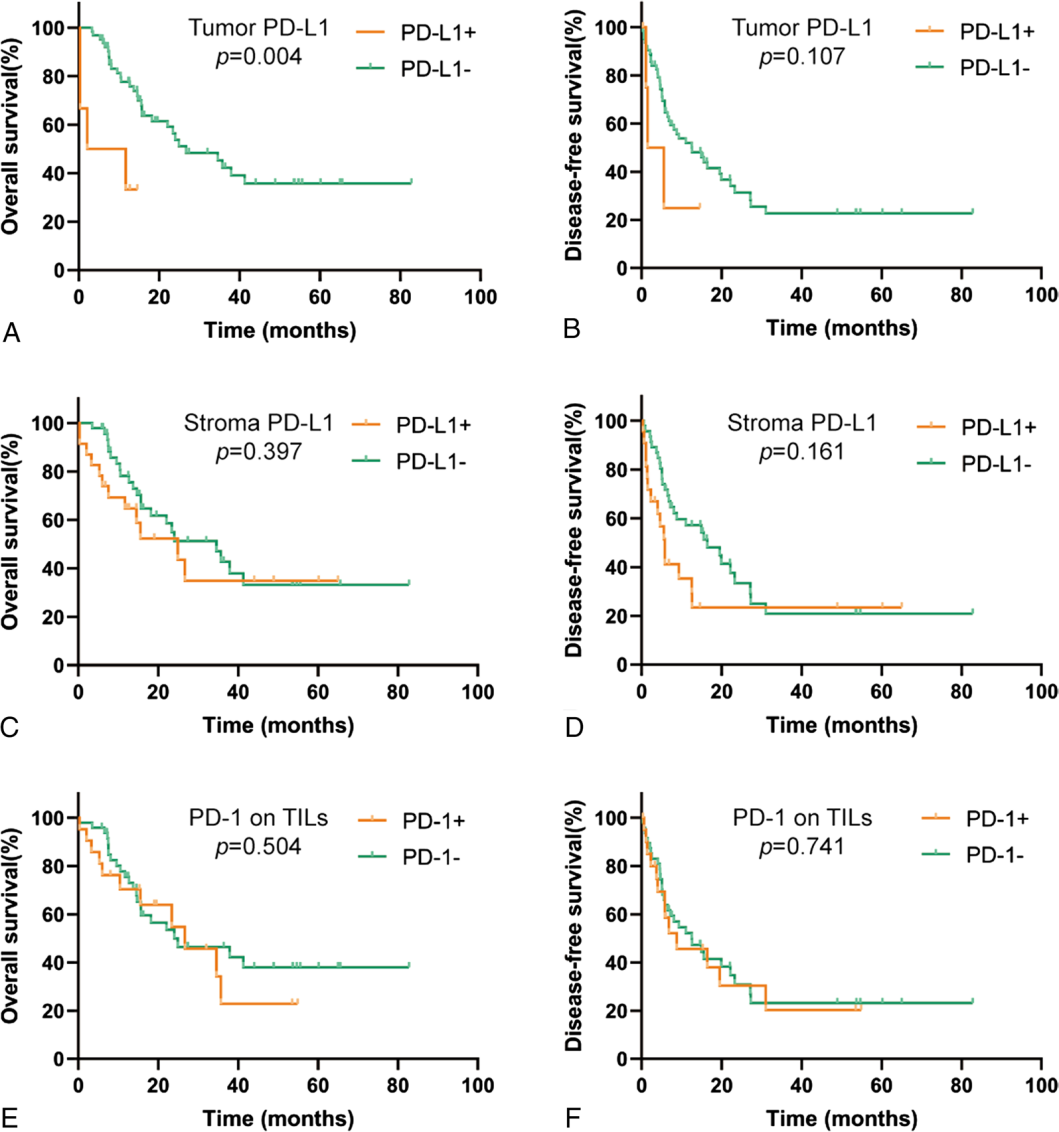
PD-L1 expression in ICC is correlated with worse OS. Association of OS and DFS with tumor PD-L1 expression (**A**, **B**) and stromal PD-L1 expression (**C**, **D**). Association of OS and DFS with PD-1 expression in TILs (**E**, **F**). The probabilities of overall and disease-free survival were estimated using the Kaplan–Meier method and compared using the log-rank statistics or the Cox proportional hazards regression model

All 69 patients completed the follow-up interviews, and 34 of them died, while 35 were alive. Overall, the median overall survival (OS) was 24 months (range:0.3–88), and disease-free median survival (DFS) was 12 months (range:0.3–88). The relationship between survival and the expression of PD-L1 or PD-1 are shown in Fig. 3. The expression of PD-L1 in cancer cells was related to OS. The overall median survival rate (6.9 months) of the patients with PD-L1-positive cancer cells was significantly poorer than that of the PD-L1-negative patients (26.7 months) (*p* = 0.004). DFS (5.8 months) of the patients with cancer cells expressing PD-L1 was also poorer than that of PD-L1-negative patients (16.4 months), but there was no statistical difference (p = 0.107). The higher the PD-L1 expression was, the worse the OS (*p* = 0.004).

**Fig. 4:**
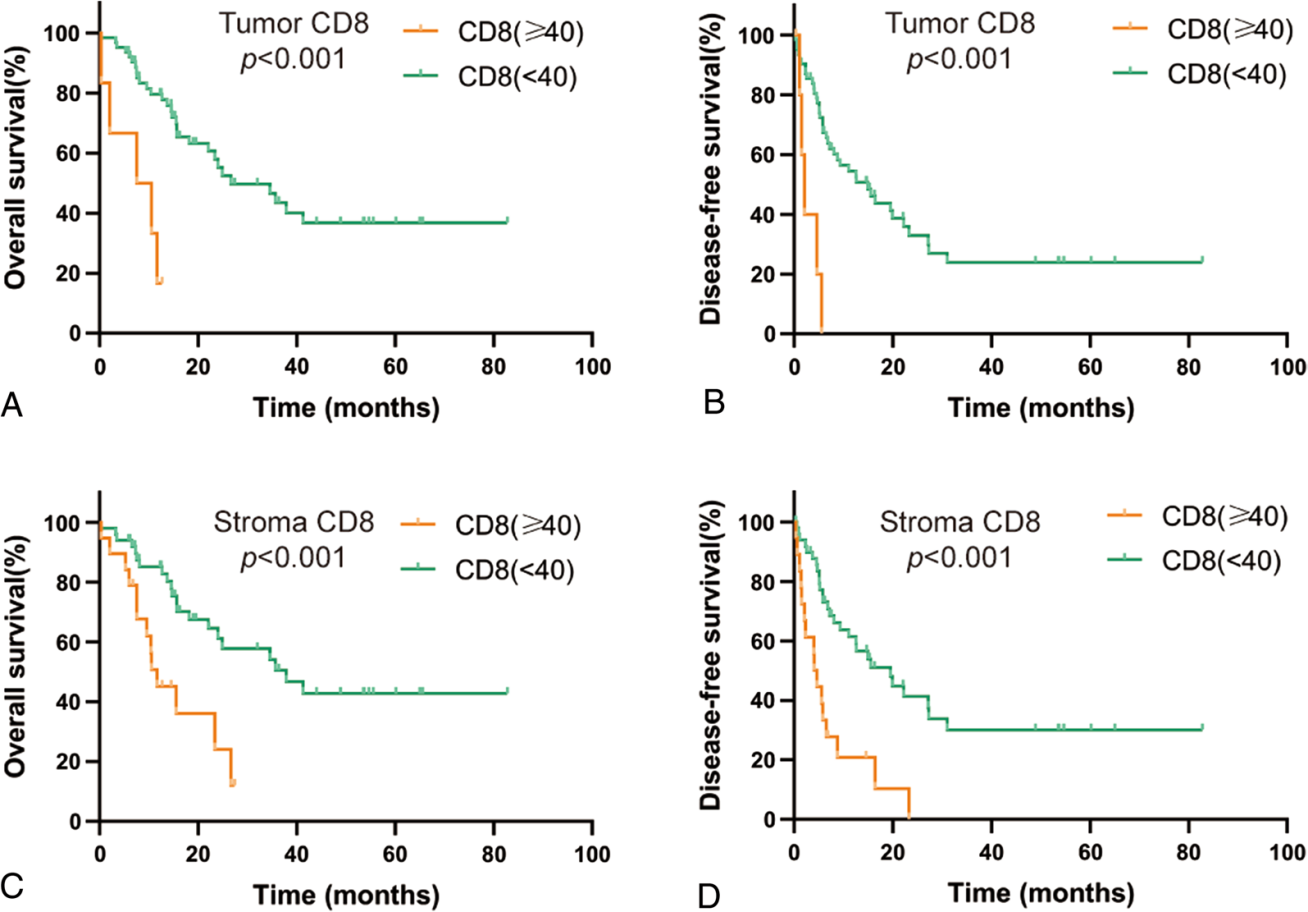
Increasing intratumoral and stromal CD8+ T-cell densities in ICC are correlated with worse OS and DFS. Association of OS and DFS with the CD8+ T-cell density within tumors (**A**, **B**) and in the stroma (**C**, **D**). Probabilities of overall and disease-free survival were estimated using the Kaplan–Meier method and compared using the log-rank statistics or the Cox proportional hazards regression model. The upper quartile was used as the breakpoint for the stromal CD8+ T-cell density, and the approximate lower and upper quartiles were used for the tumor CD8+ T-cell density. The reported HRs for the intratumoral CD8+ T-cell density reflect the survival comparison between tumors with a high density (≥ 40 CD8+ T cells/Hp) and those with a low density (< 40 CD8+ T cells/Hp)

The density of CD8+ T-cells in the tumor and stroma also correlated with OS and DFS. Results are shown in Fig. 4. The OS and DFS rates in the high CD8+ TIL and CD8+ NIL groups were significantly poorer than those in the low CD8+ TIL and NIL groups (All *p* < 0.001).

Based on the univariate analysis data shown in Table 3, the risk factors for a poorer ICC prognosis included the expression of PD-L1 in the tumor, the numbers of CD8-positive TILs and NILs, the TNM stage, and high serum levels of direct bilirubin (DB) and gamma-glutamyl transpeptidase (γ-GT). Multivariate analysis with the Cox regression model showed that the expression of PD-L1 in the tumor, the number of CD8-positive NILs and a high serum level of γ-GT were significant independent risk factors for a poorer prognosis.

**Table 3:** Univariate and multivariate analyses of prognostic factors in patients with intrahepatic cholangiocarcinoma

	Univariate analysis		Multivariate analysis	
	*p*	Exp (B)	95% CI	*p*
PD-L1 cancerous cell(+)	0.002	0.339	0.118–0.959	0.042
CD8+ TIL ≥ 40	0.009			
CD8+ NIL ≥ 40	0.002	0.382	0.147–0.994	0.048
TNM stage	0.003			
DB > 6.8umol/L	0.002			
γ-GT > 35U/L	0.006	0.252	0.083–0.759	0.014

*DB* direct bilirubin, *γ-GT* gamma-glutamyl transpeptidase

## Discussion

In this study, conclusions were drawn based on a retrospective analysis of 69 resected ICC specimens. We studied the expression of PD-L1 in ICC tumor and stromal cells, the expression of PD-1 in lymphocytes, and the density of CD8^+^ TILs and NILs. The results showed that the expression of PD-L1 in ICC cancer cells but not in stromal cells was highly correlated with a poorer prognosis. The higher density of CD8+ lymphocytes in the tumor and stroma was related to a poor prognosis, which is different from the results of a previous study [31].

PD-L1 can be secreted by both cancer cells and interstitial cells in tumors [17]. Ye et al. [31] have reported that in 31 ICC cases, no expression of PD-1 was found in tumor cells. The results were different for PD-L1 expression in ICC, and a strong expression of PD-L1 in patients was predictive of a poor prognosis. Sabbatino et al. [32] shown that the expression rate of PD-L1 in ICC tumor cells was only 29.6% (8/27), and the prognosis for these PD-L1-expressing patients was poor. Sato et al. [33] have reported a high incidence of cholangiocarcinoma in young people working at a printing plant in Osaka, Japan; the disease is related to long-term exposure to organic solvents, and the researchers classified these cases as occupational exposure-related cholangiocarcinoma. They studied the association of occupational cholangiocarcinoma with this particular location. The expression of PD-L1 in occupational cholangiocarcinoma was 100% (10/10), which was significantly higher than that in non-occupational cholangiocarcinoma (10%, 2/23), and the prognosis was poor. The results of our study showed that normal bile duct epithelial cells hardly expressed PD-L1; only 11.6% (8/69) of the samples were positive for the expression of PD-L1 in tumor cells, while the incidence of expression of PD-L1 in interstitial cells was 50.1% (35/69). Similar to the previous findings, the presence of PD-L1 expression suggested a poor prognosis for the patients, in terms of both OS or DFS. Our team’s previous research on ICC revealed that patients with PD-L1 expression level ≥ 2% had worse OS. Then we chose expression level ≥ 2% as a positive expression of PD-L1. Similar to other tumors, there are discrepancies in the PD-L1 expression rate in ICC among different investigators, which may be due to differences in the immunohistochemical antibodies, tissue samples, and immunohistochemical evaluation methods used. Thus, based on published tumor-related results, a high level of expression of PD-L1 consistently suggests a poor prognosis for the patient, and the expression of PD-L1 is a potential predictor and therapeutic target for ICC. At present, studies on the expression and mechanism of action of PD-L1 in HCC have made progress. Calderaro [27] believes that the expression of PD-L1 can reflect the clinical and pathological features of HCC, and PD-L1-positive tumors are more aggressive. In terms of the treatment, a PD-L1-targeted drug combination with sorafenib is more effective than monotherapy in treating HCC patients.

PD-1 is mainly located in the cytoplasm of lymphocytes. We divided lymphocytes into tumor-infiltrating lymphocytes (TILs) and non-tumor-infiltrating lymphocytes (NILs) according to whether PD-1-positive lymphocytes infiltrated in the tumor cell mass or not. Ye et al. have performed PD-1 immunohistochemical staining on all ICC specimens, and a large number of PD-1-positive T lymphocytes were observed in the tumor or in the stroma, whereas no PD-1 expression was observed in tumor cells. Sabbatino et al. have found that PD-1 was expressed in TILs to a different degree. Among them, TILs with low, middle, and high levels of expression accounted for 42.3% (11/26), 23.1% (6/26), and 34.6% (9/26), but there was no statistical relationship between the expression of PD-1 and OS of the patients. Sato’s results have shown that the numbers of PD-1-positive lymphocytes and CD8+ T cells infiltrating in the tumor were significantly higher in occupational cholangiocarcinoma than in non-occupational cholangiocarcinoma, but this study did not report the existence of a correlation between PD-1 and OS. We found that the rate of PD-1 expression in tumor lymphocytes was 30.4% (21/69), suggesting that the expression rate of PD-1 was not significantly different from that of ICC occurrence. Currently, there is no PD-1-targeted therapy for ICC; however, the results of some studies [30, 34] suggest that the targeted drug lenvatinib, combined with a PD-1 inhibitor, nivolumab, is superior to sorafenib monotherapy in the treatment of advanced HCC patients, which provides the directions for PD-1 drug therapy in patients with ICC.

After PD-L1 binds to PD-1, T lymphocytes are inactivated and lose their immune killing effect [17]. However, the correlation between the number of CD8-positive T cells and the expression of PD-1 or PD-L1 is not very clear. Studies by Ye et al. on ICC [31], Gabrielson et al. on HCC [35], and Thompson et al. on gastric adenocarcinoma[26] have led to similar conclusions that the expression of PD-L1 is negatively correlated with the number of CD8-positive T cells. Xie et al. [36] have found in 167 HCCs that the number of CD8+ T cells in PD-L1-positive tumors (mean 75/Hp) was much higher than that in PD-L1-negative tumors (mean 25/Hp; *p* < 0.0001). The difference is explained by the accumulation of CD8+ T cells in the tumor microenvironment, where they can stimulate the expression of PD-L1 by releasing specific factors. PD-L1 is not continuously expressed in tumor cells. Our results are similar to those of the previous study and show that patients with high CD8+ TIL and NIL counts have a poor prognosis. In PD-L1-positive patients, the numbers of CD8+ T cells, including TILs and NILs, are higher than those in PD-L1-negative patients, and the higher the number of CD8+ TILs is, the poorer the OS and DFS of the patients are. Similarly, the higher the number of CD8+ NILs (≥ 40/hp), the poorer the OS and DFS of the patients are. We hypothesized that in the cases of high expression of PD-L1 or PD-1, the number of CD8+ T cells increases not as a result of PD-L1- or PD-1-caused dysfunction but because of the compensatory mechanism, which leads to an increase in the number of CD8-positive T cells.

Numerous studies have shown that PD-1/PD-L1 are important components of the tumor immune escape [18, 37], and their role is related to the defect in T lymphocyte immune function [38, 39]. The traditional view is that the number of CD8-positive T cells in tumor tissues represents the number of active T cells and reflects the immune activity of the organism against the tumor [39, 40]. The binding of PD-L1 to PD-1 results in the inactivation of T lymphocytes and the loss of their immune killing effect, thereby promoting the development of tumors. Ye et al. [31] have discovered that the expression of PD-L1 in the tumor was negatively correlated with the number of CD8-positive T cells. Similarly, Gabrielson et al. [35] have also concluded, based on a study of 65 HCC cases, that the expression of PD-L1 in tumor tissue is negatively correlated with the number of CD8-positive T cells. However, in tumor-surrounding tissue, the expression of PD-L1 was positively associated with the number of CD8+ T cells, similar to normal liver tissue. The results may indicate that PD-L1 expression is related to the inactivation of CD8+ T cells.

However, some studies have not found a positive correlation between the number of CD8-positive T cells and the expression of PD-1 or PD-L1. Xie et al. [36] have retrospectively studied 167 cases of HCC and speculated that when CD8-positive T cells are accumulated in the tumor microenvironment, they may stimulate the expression of PD-L1 by releasing specific factors and that the expression of PD-L1 is not continuous in tumor cells. Our results showed that the numbers of CD8-positive TILs and NILs were significantly higher in PD-L1-positive tumors than in PD-L1-negative tumors, and the higher the density of CD8-positive T cells was, the poorer the OS and DFS of the patient were. Therefore, we hypothesized that the number of CD8-positive T cells does not directly represent the number of active T cells, especially in PD-1- or PD-L1-positive tumors, and that CD8-positive T cells may be in a hypofunctional state. Based on the multivariate analysis data, the expression of PD-L1 in the tumor, the number of CD8-positive NILs and a high serum level of γ-GT are independent risk factors for a worse prognosis of ICC.

Underlying liver diseases may affect the PD-1/PD-L1 expression. Wang et al. [29] have studied the HCC pathology and found that the PD-1 expression is associated with the viral load in HBV liver infection. The higher the HBV viral load is in the serum, the higher the positive rate of PD-1 is in liver tissue. Zhang et al. [41] have shown that increased expression of PD-1 may inhibit immunity, which is beneficial for viral replication, prolongs the course of chronic hepatitis B and promotes the progression of hepatitis B to liver cirrhosis. Our study did not show a significant correlation between HBV infection and the expression of PD-1 or PD-L1 in the tumor. However, the incidence of background hepatic fibrosis or cirrhosis was significantly higher in the PD-L1-positive ICC patients than in the PD-L1-negative group. In addition, there was a relationship between clinicopathological features and PD-1/PD-L1 expression. Ye’s [31] results have shown poorer ICC differentiation at a later TNM stage, which was related to a high expression of PD-L1 in ICC cells. Sabbatino et al. [32] have also reported that increased expression of PD-L1 was associated with an earlier T stage. Our study also showed that the volume of PD-1-positive tumors was significantly smaller than that of PD-1-negative tumors. PD-1-positive tumors were more likely to lead to MVI.

The main limitations of this study were the small number of ICC cases with a complete follow-up, due to a low incidence, a low resection rate of ICC tumors, single-center experience and that the patients were scattered over a large geographic area. In addition, no detection of CD8+ T cell activity was performed in this study. In addition, because of the use of different antibodies for immunohistochemistry and different individual judgment standards, the results from different research centers are not comparable.

## Conclusions

PD1/PD-L1-targeted immunotherapy is the most promising treatment option for improving the survival of ICC patients. The results of clinical trials of anti-PD-1 drugs in HCC patients are very satisfactory, making the prospects for immunotherapy of liver malignancies brighter. However, our results suggest that high expression of PD-L1 in tumor cells in ICC is associated with an elevated CD8+ TIL density and a poor prognosis. The specific mechanism remains to be elucidated. Evaluation of ICC patients by immunohistochemistry and other methods before treatment may help develop individualized approaches to the treatment with PD-1/PD-L1 inhibitors.

## Data Availability

The data that support the findings of our research are available from Nanjing Drum Tower Hospital, but restrictions apply to the availability of these data, which were used under license for the current study, and so are not publicly available.

## Abbreviations

ICC: Intrahepatic cholangiocarcinoma
HCC: Hepatocellular carcinoma
PD-L1: Programmed death-ligand 1
PD-1: Programmed death factor 1
TIL: Tumor infiltrating lymphocytes
NIL: Nontumor infiltrating lymphocytes
HBV: Hepatitis B virus
HCV: Hepatitis C virus
HBsAg: Hepatitis B surface antigen
Hp: High power lens
CA19-9: Carbohydrate antigen 19-9
TB: Total bilirubin
DB: Direct bilirubin
ALT: Alanine transaminase
AST: Aspartate transaminase
γ-GT: Gamma-glutamyl transpeptidase
MF: Mass-forming
CLC: Cholangiolocellular carcinoma
cICC: Conventional intrahepatic cholangiocarcinoma
MVI: Microvascular invasion
AJCC: American Joint Committee on Cancer
UICC: Union for International Cancer Control
OS: Overall survival
DFS: Disease-free survival

## References

[1] Valle JW, Kelley RK, Nervi B, Oh DY, Zhu AX (2021). Biliary tract cancer. Lancet.

[2] Razumilava N, Gores GJ (2014). Cholangiocarcinoma. Lancet.

[3] Bridgewater J, Galle PR, Khan SA, Llovet JM, Park JW, Patel T (2014). Guidelines for the diagnosis and management of intrahepatic cholangiocarcinoma. J Hepatol.

[4] DeOliveira ML, Cunningham SC, Cameron JL, Kamangar F, Winter JM, Lillemoe KD (2007). Cholangiocarcinoma: thirty-one-year experience with 564 patients at a single institution. Ann Surg.

[5] Bergquist A, von Seth E (2015). Epidemiology of cholangiocarcinoma. Best Pract Res Clin Gastroenterol.

[6] Komuta M, Govaere O, Vandecaveye V, Akiba J, Van Steenbergen W, Verslype C (2012). Histological diversity in cholangiocellular carcinoma reflects the different cholangiocyte phenotypes. Hepatology.

[7] Tyson GL, El-Serag HB (2011). Risk factors for cholangiocarcinoma. Hepatology.

[8] Kim HJ, Kim JS, Joo MK, Lee BJ, Kim JH, Yeon JE (2015). Hepatolithiasis and intrahepatic cholangiocarcinoma: a review. World J Gastroenterol.

[9] Li H, Hu B, Zhou ZQ, Guan J, Zhang ZY, Zhou GW (2015). Hepatitis C virus infection and the risk of intrahepatic cholangiocarcinoma and extrahepatic cholangiocarcinoma: evidence from a systematic review and meta-analysis of 16 case-control studies. World J Surg Oncol.

[10] Vogel A, Wege H, Caca K, Nashan B, Neumann U (2014). The diagnosis and treatment of cholangiocarcinoma. Dtsch Arztebl Int.

[11] Ruzzenente A, Conci S, Valdegamberi A, Pedrazzani C, Guglielmi A (2015). Role of surgery in the treatment of intrahepatic cholangiocarcinoma. Eur Rev Med Pharmacol Sci.

[12] Mavros MN, Economopoulos KP, Alexiou VG, Pawlik TM (2014). Treatment and prognosis for patients with intrahepatic cholangiocarcinoma: systematic review and meta-analysis. JAMA Surg.

[13] Moeini A, Sia D, Bardeesy N, Mazzaferro V, Llovet JM (2016). Molecular pathogenesis and targeted therapies for intrahepatic cholangiocarcinoma. Clin Cancer Res.

[14] Xie D, Ren Z, Fan J, Gao Q (2016). Genetic profiling of intrahepatic cholangiocarcinoma and its clinical implication in targeted therapy. Am J Cancer Res.

[15] Patel T (2011). Cholangiocarcinoma–controversies and challenges. Nat Rev Gastroenterol Hepatol.

[16] Sirica AE, Gores GJ, Groopman JD, Selaru FM, Strazzabosco M, Wei Wang X (2019). Intrahepatic cholangiocarcinoma: continuing challenges and translational advances. Hepatology.

[17] Topalian SL, Drake CG, Pardoll DM (2012). Targeting the PD-1/B7-H1(PD-L1) pathway to activate anti-tumor immunity. Curr Opin Immunol.

[18] Afreen S, Dermime S (2014). The immunoinhibitory B7–H1 molecule as a potential target in cancer: killing many birds with one stone. Hematol Oncol Stem Cell Ther.

[19] Okazaki T, Honjo T (2007). PD-1 and PD-1 ligands: from discovery to clinical application. Int Immunol.

[20] Gani F, Nagarajan N, Kim Y, Zhu Q, Luan L, Bhaijjee F (2016). Program death 1 immune checkpoint and tumor microenvironment: implications for patients with intrahepatic cholangiocarcinoma. Ann Surg Oncol.

[21] Spranger S, Spaapen RM, Zha Y, Williams J, Meng Y, Ha TT (2013). Up-regulation of PD-L1, IDO, and T(regs) in the melanoma tumor microenvironment is driven by CD8(+) T cells. Sci Transl Med.

[22] Wu C, Zhu Y, Jiang J, Zhao J, Zhang XG, Xu N (2006). Immunohistochemical localization of programmed death-1 ligand-1 (PD-L1) in gastric carcinoma and its clinical significance. Acta Histochem.

[23] Donnem T, Hald SM, Paulsen EE, Richardsen E, Al-Saad S, Kilvaer TK (2015). Stromal CD8+ T-cell density—a promising supplement to TNM staging in non-small cell lung cancer. Clin Cancer Res.

[24] Gajewski TF, Schreiber H, Fu YX (2013). Innate and adaptive immune cells in the tumor microenvironment. Nat Immunol.

[25] Rosenbaum MW, Bledsoe JR, Morales-Oyarvide V, Huynh TG, Mino-Kenudson M (2016). PD-L1 expression in colorectal cancer is associated with microsatellite instability, BRAF mutation, medullary morphology and cytotoxic tumor-infiltrating lymphocytes. Mod Pathol.

[26] Thompson ED, Zahurak M, Murphy A, Cornish T, Cuka N, Abdelfatah E (2017). Patterns of PD-L1 expression and CD8 T cell infiltration in gastric adenocarcinomas and associated immune stroma. Gut.

[27] Calderaro J, Rousseau B, Amaddeo G, Mercey M, Charpy C, Costentin C (2016). Programmed death ligand 1 expression in hepatocellular carcinoma: relationship With clinical and pathological features. Hepatology.

[28] Gao Q, Wang XY, Qiu SJ, Yamato I, Sho M, Nakajima Y (2009). Overexpression of PD-L1 significantly associates with tumor aggressiveness and postoperative recurrence in human hepatocellular carcinoma. Clin Cancer Res.

[29] Wang BJ, Bao JJ, Wang JZ, Wang Y, Jiang M, Xing MY (2011). Immunostaining of PD-1/PD-Ls in liver tissues of patients with hepatitis and hepatocellular carcinoma. World J Gastroenterol.

[30] Wang Y, Li H, Liang Q, Liu B, Mei X, Ma Y (2015). Combinatorial immunotherapy of sorafenib and blockade of programmed death-ligand 1 induces effective natural killer cell responses against hepatocellular carcinoma. Tumour Biol.

[31] Ye Y, Zhou L, Xie X, Jiang G, Xie H, Zheng S (2009). Interaction of B7–H1 on intrahepatic cholangiocarcinoma cells with PD-1 on tumor-infiltrating T cells as a mechanism of immune evasion. J Surg Oncol.

[32] Sabbatino F, Villani V, Yearley JH, Deshpande V, Cai L, Konstantinidis IT (2016). PD-L1 and HLA Class I antigen expression and clinical course of the disease in intrahepatic cholangiocarcinoma. Clin Cancer Res.

[33] Sato Y, Kinoshita M, Takemura S, Tanaka S, Hamano G, Nakamori S (2017). The PD-1/PD-L1 axis may be aberrantly activated in occupational cholangiocarcinoma. Pathol Int.

[34] El-Khoueiry AB, Sangro B, Yau T, Crocenzi TS, Kudo M, Hsu C (2017). Nivolumab in patients with advanced hepatocellular carcinoma (CheckMate 040): an open-label, non-comparative, phase 1/2 dose escalation and expansion trial. Lancet.

[35] Gabrielson A, Wu Y, Wang H, Jiang J, Kallakury B, Gatalica Z (2016). Intratumoral CD3 and CD8 T-cell densities associated with relapse-free survival in HCC. Cancer Immunol Res.

[36] Xie QK, Zhao YJ, Pan T, Lyu N, Mu LW, Li SL (2016). Programmed death ligand 1 as an indicator of pre-existing adaptive immune responses in human hepatocellular carcinoma. Oncoimmunology.

[37] Patel SP, Kurzrock R (2015). PD-L1 expression as a predictive biomarker in cancer immunotherapy. Mol Cancer Ther.

[38] Saresella M, Rainone V, Al-Daghri NM, Clerici M, Trabattoni D (2012). The PD-1/PD-L1 pathway in human pathology. Curr Mol Med.

[39] Bhaijee F, Anders RA (2015). PD-L1 expression as a predictive biomarker: is absence of proof the same as proof of absence. JAMA Oncol.

[40] Hamanishi J, Mandai M, Iwasaki M, Okazaki T, Tanaka Y, Yamaguchi K (2007). Programmed cell death 1 ligand 1 and tumor-infiltrating CD8+ T lymphocytes are prognostic factors of human ovarian cancer. Proc Natl Acad Sci U S A.

[41] Zhang G, Li N, Zhang P, Li F, Yang C, Zhu Q (2014). PD-1 mRNA expression is associated with clinical and viral profile and PD1 3'-untranslated region polymorphism in patients with chronic HBV infection. Immunol Lett.

